# Meet the neighbors: Mapping local protein interactomes by proximity‐dependent labeling with BioID

**DOI:** 10.1002/pmic.201600123

**Published:** 2016-07-27

**Authors:** Renata Varnaitė, Stuart A. MacNeill

**Affiliations:** ^1^ School of BiologyUniversity of St AndrewsNorth HaughSt AndrewsScotland, UK

**Keywords:** Biotinylation, Interactome, Promiscuous biotin ligase (BirA*), Protein–protein interactions, Proximity‐dependent biotin identification, Technology

## Abstract

Proximity‐dependent biotin identification (BioID) is a recently developed method that allows the identification of proteins in the close vicinity of a protein of interest in living cells. BioID relies on fusion of the protein of interest with a mutant form of the biotin ligase enzyme BirA (BirA*) that is capable of promiscuously biotinylating proximal proteins irrespective of whether these interact directly or indirectly with the fusion protein or are merely located in the same subcellular neighborhood. The covalent addition of biotin allows the labeled proteins to be purified from cell extracts on the basis of their affinity for streptavidin and identified by mass spectrometry. To date, BioID has been successfully applied to study a variety of proteins and processes in mammalian cells and unicellular eukaryotes and has been shown to be particularly suited to the study of insoluble or inaccessible cellular structures and for detecting weak or transient protein associations. Here, we provide an introduction to BioID, together with a detailed summary of where and how the method has been applied to date, and briefly discuss technical aspects involved in the planning and execution of a BioID study.

**Abbreviations**
AC: apical cap
APEX: ascorbate peroxidase
BioID: proximity‐dependent biotin identification
CCM: cerebral cavernous malformation
CDPK: calcium‐dependent protein kinase
EBs: elementary bodies
FAZ: flagellum attachment zone
HIV‐1: human immunodeficiency virus‐1
IMC: inner membrane complex
INM: inner nuclear membrane
LaA: lamin A
LMP: latent membrane protein
NE: nuclear envelope
NLS: nuclear localization signal
NMD: nonsense‐mediated mRNA decay
NPC: nuclear pore complex
PCM: pericentriolar material
PP2A: phosphoprotein phosphatase 2A
RBs: reticulate bodies
SFN: stratifin
TRAPP: trafficking protein particle

## 1 Introduction

Building comprehensive protein interactome maps is key to understanding cell function. A particular challenge is in building maps that account not only for direct binary protein–protein interactions, but also which incorporate information on indirect interactions (for example, between proteins that do not interact directly with one another, but which are components of the same multiprotein complex) and proximal protein networks. Interactome discovery methods also need to take into account the dynamic nature of many cellular processes and overcome the technical challenges posed by weak protein–protein interactions.

**Figure 1:**
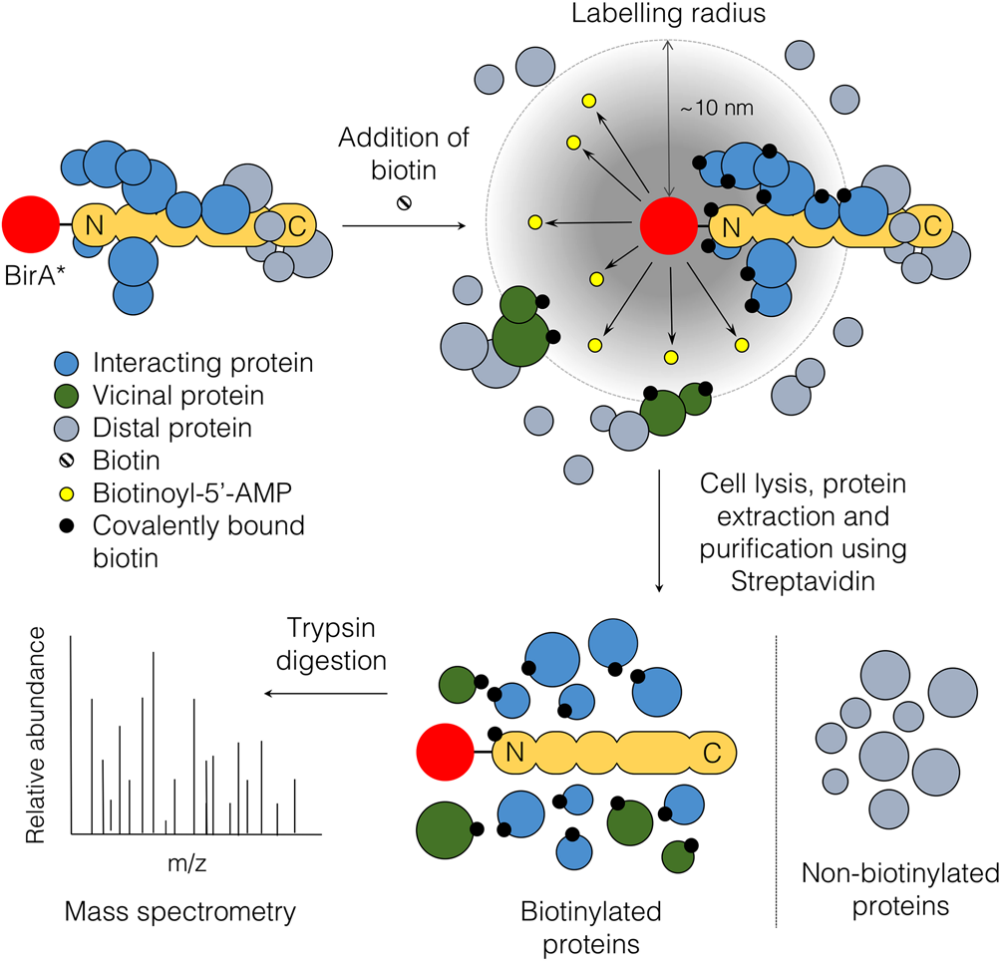
BioID. The protein of interest is fused with a promiscuous form (BirA*) of the bacterial biotin ligase BirA and expressed in cells. In the figure, the target protein (pale orange) has an elongated structure and BirA* (red) is shown fused to the *N*‐terminal end (*C*‐terminal fusion is also possible). BirA* converts exogenously added free biotin to highly reactive but labile biotinyl‐5′‐AMP (yellow) which is released from the enzyme's active site allowing it to react with primary amines on proximal proteins (blue, green), irrespective of whether they interact (directly or indirectly via additional protein–protein interactions) with the fusion protein or are merely in the close vicinity (labeling radius ∼10 nm, based on studies of the NPC Y‐complex, see text for details). Distal proteins (gray), whether they interact with the BirA* fusion or not, are not labeled. In the example shown, with the BirA* enzyme fused to the *N*‐terminal end of the protein of interest, note how factors that interact at the *C*‐terminal end of the protein are not labeled, as they lie beyond the effective labeling radius. Following biotin labeling, cells are lysed and proteins extracted under relatively harsh conditions. Biotinylated proteins are then purified using streptavidin (nonbiotinylated proteins are discarded) and identified by MS. See text for details and references.

Proximity‐dependent labeling by modifying enzymes 1, 2 is a novel approach for protein–protein interaction screening that addresses these challenges. When fused to the protein of interest the modifying enzymes covalently attach a tag on proximal and potentially interacting proteins, which allows their subsequent purification and identification by MS. Examples of such techniques include selective proteomic proximity labeling using tyramide 3, ascorbate peroxidase (APEX) 4, 5, and proximity‐dependent biotin identification (BioID) 6. Here we focus on BioID, which is already making a major impact on our understanding of cell structure and function 7. BioID offers significant advantages over conventional interactome discovery methods, particularly in regards to the identification of transient or weak interactions but also through its applicability to insoluble subcellular structures. The BioID technique makes use of the ability of a mutant form of the *Escherichia coli* biotin ligase (BirA*) to promiscuously biotinylate proximal proteins 6. Biotin ligases are highly conserved and biologically important enzymes that facilitate the attachment of biotin to their target proteins 8. The well‐characterized wild‐type biotin ligase from *E. coli* has a single protein target, a subunit of acetyl‐CoA carboxylase. Biotinylation is a two‐step reaction, where the biotin ligase uses biotin and ATP (adenosine triphosphate) to generate a highly reactive biotinoyl‐5′‐AMP (adenosine monophosphate) intermediate 9, which later reacts with a specific lysine on the target protein to form an amide bond between biotin and the lysine side chain, releasing AMP 8. The wild‐type biotin ligase has high affinity for the biotinoyl‐5′‐AMP intermediate, which remains within the active site until the enzyme recognizes its acetyl‐CoA carboxylase substrate. In contrast, the mutant form of *E. coli* biotin ligase BirA*, which carries R118G mutation at the active site, has significantly reduced affinity for biotinoyl‐5′‐AMP and releases it prematurely 10, 11. In this way, the highly reactive biotinoyl‐5′‐AMP can react with accessible lysine side chains on the surrounding proteins in a proximity‐dependent manner, leaving the proteins covalently marked with biotin. This promiscuous covalent labeling of proximal proteins with biotin forms the basis of BioID method (Fig. 1) 6. In a typical BioID experiment, a gene fusion encoding the protein of interest and BirA* is expressed in vivo. Once synthesized, the protein of interest therefore carries the BirA* tag (35.4 kDa) on either its *N*‐ or *C*‐terminus. Biotin is then added and after a suitable incubation period (typically 24 h) the cells are harvested, proteins extracted, and biotinylated proteins purified using streptavidin. MS is then used to identify the biotinylated proteins.

As labeling is proximity‐dependent, potential interactors of the protein of interest must reside within the area reachable by the reactive biotinoyl‐5′‐AMP. This area can be described as the practical labeling radius of BirA*. Initial studies by Roux et al. 6 suggested the radius to be approximately 20–30 nm. This was later reduced to ∼10 nm by using the known structure of the nuclear pore complex (NPC) as a “molecular ruler” 12. Although the exact value may vary depending on the protein complex under study, this radius is sufficient to allow detection of both direct interactors and noninteracting vicinal proteins.

BioID has several key advantages over more established interactome discovery methods 6. First, it is important to emphasize that while the biotin labeling is proximity‐dependent, it does not require either direct or indirect protein–protein interaction between the BirA* fusion and the protein that becomes biotinylated (illustrated in Fig. 1). Noninteracting but proximal proteins are also labeled, giving valuable information on the proteomic landscape surrounding the target. Second, proteins that transiently interact with—or are transiently proximal to—the BirA* fusion will also be biotinylated and, crucially, the biotinylation mark will remain even after the interaction has ceased or the protein has moved out of the vicinity of the BirA* fusion (Fig. 1). BioID is therefore well suited to studying cellular processes that are dynamic in space and time. Third, unlike more conventional methods such as affinity purification, where stringent extraction conditions will disrupt protein–protein interactions and result in data loss, in BioID cell lysis and subsequent isolation of biotinylated proteins via streptavidin binding can be performed under stringent conditions. For this reason, BioID is proving particularly useful for investigation of insoluble proteins structures such as nuclear lamina or centrosome. Finally, the only requirement for BioID is expression of a correctly localized BirA* fusion protein. It is not absolutely essential that the fusion protein is expressed at an appropriate level (comparable to the endogenous target protein) or is functional, although as discussed below (see Section 5) there may be advantages to ensuring that these conditions are met.

While proximity‐dependent labeling techniques have been reviewed in the past 1, 7, here we focus exclusively on BioID. We provide a comprehensive summary of how the method has been used to map local interactomes in a variety of cellular processes, both in mammals and in unicellular eukaryotes, before discussing technical aspects involved in the planning and execution of a BioID study and looking ahead to future developments.

## 2 Application of BioID in mammalian cells

### 2.1 Nuclear lamina

BioID was first introduced in 2012 in a landmark paper by Roux et al. 6 reporting the application of the method to the lamin A (LaA) protein, a key constituent of the nuclear lamina, the filamentous protein network that underlies the inner nuclear membrane (INM) 13. The lamina is made up of lamins (both A‐ and B‐types) and nuclear lamin‐associated membrane proteins, such as LAP1 (lamin‐associated polypeptide 1), LAP2, and emerin, and acts as an anchor for NPCs that span the nuclear envelope (NE). Lamins are intermediate filament proteins 14 and are highly insoluble, making them largely intractable to conventional methods for screening for protein–protein interactions and thus an ideal system to test the effectiveness of the BioID concept.

To apply BioID to LaA, Roux et al. 6 generated HEK293 cells that stably and inducibly expressed a Myc‐epitope tagged BirA*‐LaA fusion protein. Like the endogenous LaA, the fusion localizes predominantly to the NE but with a subpopulation present throughout the nucleus, indicating that addition of the Myc‐BirA* moiety to the *N*‐terminus of the LaA protein does not perturb its localization, an important precondition for BioID. Following addition of 50 μM biotin to the culture medium for 24 h, Myc‐BirA*‐LaA expressing cells were lysed and proteins extracted under denaturing conditions, with parental HEK293 cells being processed in parallel. Biotinylated proteins were then captured using streptavidin and subjected to MS.

Over 120 proteins were identified as being proximal to the Myc‐BirA*‐LaA fusion in this study, including a number of previously characterized NE components, including the known lamin interactors LAP1, LAP2, emerin, and MAN1 14. Twelve proteins associated with nuclear‐cytoplasmic transport were also detected, including the NPC components Nup50, Nup153, and ELYS 15. A previously uncharacterized protein FAM169A (later renamed SLAP75) was also identified and was shown to be a novel NE constituent by immunofluorescence microscopy, elegantly demonstrating the power of the BioID approach for identifying novel proteins 6. A further ten uncharacterized proteins were also identified in this screen: whether these, like FAM169A/SLAP75, will also be constituents of the NE remains to be seen.

In a second study focused on the nuclear lamina, Dressen and colleagues 16 used BioID to compare the interactomes of LaA and progerin, a truncated form of the LaA protein that is responsible for the progressive aging syndrome Hutchinson–Gilford progeria. While the two interactomes displayed considerable overlap, in experiments performed in both primary fibroblasts and pluripotent ES cells, significantly less LAP2 was biotinylated by Myc‐BirA*‐progerin compared to the full‐length Myc‐BirA*‐LaA, suggesting a possible mechanism for how the LaA truncation causes Hutchinson–Gilford progeria 16.

More recently, BioID has also been used to generate an interactome for lamin B1 (LMNB1). In these experiments 17 LMNB1 was expressed as a Myc‐MirA*‐LMNB1 fusion from a lentiviral vector in the mouse hepatocyte cell line AML12. Over 180 potential interactors were identified in this screen, including LaA, MAN1, LAP2 and, unexpectedly, the variant histone macroH2A1 (mH2A1) previously shown to be associated with repressive chromatin. Reciprocol BioID using a Myc‐BirA*‐mH2A1 fusion led to biotinylation LMNB1, confirming the spatial proximity of these factors.

### 2.2 Nuclear pore complex

Embedded in the NE, the NPC allows bidirectional transport of molecules (RNA, proteins, carbohydrates, signaling molecules, and lipids) between the nucleus and cytoplasm. The NPC is an extremely stable macromolecular structure consisting of multiple copies of ∼30 different proteins and with a molecular mass of ∼125 MDa 15.

In a second landmark paper 12, Roux and colleagues used the NPC as a model system to probe the limits of the BioID method in analyzing the architecture of large multiprotein complexes, in particular to determine the effective labeling radius of the BirA* enzyme. To achieve this, BioID was performed on components of two NPC subcomplexes, the Y‐complex (also known as the Nup107–160 complex) and the Nup93 complex. The Y‐complex is extremely stable and has an elongated Y‐shaped structure, 40 nm long and 20 nm wide, onto which individual subunits have been mapped by a variety of approaches 18. This allowed the Y‐complex to be used as a "molecular ruler" by Kim et al. 12 who placed the BirA* moiety at different defined locations on the complex, measured the extent of biotinylation of the other subunits and correlated this with distance.

For this work, HEK293 cells constitutively expressing five Y‐complex components as BirA* fusions were constructed: four proteins (Nup85, Nup107, Nup133, and Nup160) were *N*‐terminally tagged with Myc‐BirA* and one (Nup43) *C*‐terminally tagged with BirA*‐HA 12. For analysis of the Nup93 complex, a strain expressing a Myc‐BirA*‐Nup53 fusion was created. In each case, care was taken to identify clones expressing low levels of fusion protein, to prevent potential complications caused by high levels of mislocalized proteins. Prior to biotin labeling and further analysis, immunofluorescence microscopy was used to confirm that the fusion proteins largely localized with NPCs. Identification of biotinylated proteins revealed that most were known NPC components. Importantly, the Y‐complex and Nup53 fusions labeled largely nonoverlapping sets of proteins, indicative of the spatial specificity of BioID, with the Y‐complex fusions identifying other Y‐complex components and the Nup53 fusion labeling other components of the Nup93 complex and its near neighbors. By analyzing the results obtained in relation to the subunit structure of the Y‐complex, Roux and colleagues 12 estimate the practical labeling radius in their experiments to be ∼10 nm, with labeling efficiency being significantly reduced beyond this distance. Whether this value is applicable more generally (i.e. beyond the Y‐complex) remains to be seen. It should also be noted that a lack of suitably positioned lysine side chains may mean that proteins within the practical labeling radius remain unlabeled and that negative results should therefore be treated with caution. Likewise, a lack of suitably positioned lysine side chains in proteins further from BirA* many mean that the 10 nm estimate for the Y‐complex is an underestimate.

### 2.3 Centrosome and centrosome‐cilium interface

The centrosome is the principal microtubule organizing center in animal cells and comprises two cylindrical centrioles surrounded by an amorphous mass of pericentriolar material (PCM) 19. In addition to coordinating microtubule organization, centrioles also act as cilial basal bodies. Three separate studies have now appeared describing the application of BioID to probe the protein composition of the mammalian centrosome and the centrosome‐cilium interface 20, 21, 22.

Initially, Pelletier and colleagues 22 performed BioID with a Flag‐BirA*‐CEP120 fusion protein stably and inducibly expressed in HEK293 cells. CEP120 has an important role in centriole biogenesis and BioID identified a range of known centrosomal proteins proximal to it, including SPICE1 and CPAP (whose interactions with CEP120 formed the main focus of the study) as well as CEP55, CEP95, CEP97, CEP110, CEP350, and others. A number of previously uncharacterized proteins were also identified. Note that in addition to confirming that Flag‐BirA*‐CEP120 was correctly localized to the centrosomes, the function of the fusion protein (determined by its ability to rescue cells in which siRNA knockdown of CEP120 levels led to centriole overduplication) was also confirmed prior to carrying out BioID. As discussed below (Section 5), controls of this type have been performed only rarely in the BioID studies described in this review.

Centrosome duplication occurs once per cell cycle 19. In an effort to identify regulators of this process, Firat‐Karalar and Stearns 23 performed BioID on eight proteins in total. Initially, PLK4 and CEP192 (expressed as Myc‐BirA*‐PLK4 and Myc‐BirA*‐CEP192 fusions in HEK293T cells) were used. The PLK4 and CEP192 proteins are known to interact with one another but in these experiments BioID was performed with a non‐PLK4‐interacting CEP192 isoform. Both fusion proteins localized correctly to the centrosome in control experiments and expression of the Myc‐BirA*‐PLK4 protein led to formation of multiple centrioles, indicating that the protein retains at least some functionality, as a similar phenotype is seen with wild‐type PLK4 expression. Following biotin labeling, sucrose gradient centrifugation was used to enrich for centrosomes prior to protein extraction, streptavidin purification, and analysis by MS.

Among the interactors identified by BioID with Myc‐BirA*‐PLK4 were CEP152 and CEP192 (both of which had previously been shown to interact with PLK4 by other methods), STIL (a protein important for centriole duplication not previously known to be proximal to PLK4) and various other factors that had not been implicated previously in centriole duplication. The list of interactors for Myc‐BirA*‐CEP192 partially overlapped that for PLK4 and included the native HEK293 PLK4‐interacting isoform of CEP192 itself (implying that the CEP192 protein is present in multiple copies in the centrosome) as well as proteins involved in recruitment of PCM such as NEDD1 and the Aurora kinase AURKA 23.

Following on from this, Firat‐Karalar and Stearns 23 extended their analysis with *N*‐terminal Myc‐BirA* fusions of CEP152, CPAP, CEP63 and the CEP63 paralogue CCDC67, as well as a *C*‐terminal BirA* fusion of CEP152, reasoning that the elongated structure of this protein might result in spatially distinct *N*‐ and *C*‐terminal interactomes being labeled with biotin. All five proteins were shown to localize correctly prior to BioID and the CPAP protein was shown to retain at least some function, as expression of the Myc‐BirA*‐CPAP fusion led to centriole elongation as previously seen with wild‐type CPAP. BioID identified an extensive network of proximal interactions involving these proteins and revealed a previously unseen connection between centriole duplication proteins and centriolar satellites, specialized structures that appear to play a role in protein trafficking to the PCM 23. Additional BioID experiments were performed with the CCDC14 and KIAA0753 proteins to confirm this connection and to probe further the protein composition of the satellites.

The third BioID study focusing on centrosome biology is the most comprehensive to be published to date 20. In this, 58 proteins were expressed as BirA* fusions for BioID, including proteins previously found to localize to the centriole (including CEP120, CEP63, and CPAP mentioned above), to centriolar satellites (including KIAA0753), to centriolar appendages and to the centrosome‐cilium interface. As in previous studies, the BirA* fusions were stably and inducibly expressed in HEK293 cells. The resulting interactome map contains 1700 unique components in a network with >7000 potential interactions. Further investigation of a number of the previously uncharacterized proteins identified by BioID confirmed these as novel centrosome, centriolar satellite, or centriolar appendage components, and systematic functional analysis of 500 proteins (∼30% of the interactome) using siRNA and automated high‐resolution microscopy provided compelling evidence for roles of many of these proteins in centriole amplification, ciliogenesis, and centriolar satellite formation.

### 2.4 Cell junctions

Cell junctions are large multiprotein assemblies that function to mediate cell–cell and cell–matrix contacts in multicellular organisms. Three major types of cell junctions exist in mammalian cells: occluding junctions (including tight junctions, see below), communicating junctions, and anchoring junctions (including adherens junctions).

Three successive BioID studies by Anderson and colleagues 24, 25, 26 have provided significant insights into the protein composition and organization of mammalian cell junctions. Four junction proteins have been used as BirA* fusions in these studies: ZO‐1 26, E‐cadherin 25, occludin, and claudin‐4 24. ZO‐1 is a tight junction scaffold protein of >200 kDa molecular mass and a multidomain structure. The *N*‐terminal half‐containing three PDZ domains (responsible for binding to claudins, ZO‐2, and JAM1 proteins, respectively), an SH3 domain, and a guanylate kinase homology domain that binds to occludin. The *C*‐terminal half‐contains binding sites for actin filaments and the adaptor protein cingulin. For BioID, the human ZO‐1 protein was tagged at *N*‐terminal end to generate a Myc‐BirA*‐ZO‐1 fusion and separately at the *C*‐terminal end to produce a ZO‐1‐BirA*‐Myc fusion 26. Both fusions were stably and inducibly expressed in MDCK II canine kidney epithelial cells and both colocalized with endogenous canine ZO‐1 at tight junctions. BioID was performed with Myc‐BirA* expressing cells as a control and identified >400 proteins that appear to be proximal to ZO‐1, including a relatively small number of proteins previously shown to interact with ZO‐1 such as actin, cingulin, occludin, claudin‐2 and claudin‐3, as well as a large number of proteins involved in apparently unrelated cellular processes or with no known cellular function. Most interactors were labeled with both *N*‐ and *C*‐terminal ZO‐1 BirA* fusions but some were only detected with either the *N*‐ or the *C*‐terminal fusion; others were more abundant when labeled with one fusion over the other, with the *N*‐terminal Myc‐BirA*‐ZO‐1 fusion labeling a higher number of previously characterized tight junction proteins and the *C*‐terminal ZO‐1‐BirA*‐Myc fusion labeling fewer tight junction proteins and a slightly higher number of cytoskeletal factors 26. For specific proteins, the pattern of labeling matched expectations based on previous knowledge of their localization with the *N*‐ or *C*‐terminal halves of ZO‐1. Labeling of ZO‐2, for example, which is known to interact with the second of three PDZ domains in the *N*‐terminal half of ZO‐1, was much stronger with the *N*‐terminal BirA* fusion than the *C*‐terminal one, underlining the extent to which the BioID method can offer valuable information on the spatial distribution of proximal proteins in addition to a more straightforward list of the same.

Following on from their analysis of ZO‐1, Anderson and colleagues have also applied BioID to the cell adhesion protein E‐cadherin 25 and in a separate study, occludin and claudin‐4 24. For E‐cadherin, the human protein was stably and inducibly expressed as an *N*‐terminal E‐CAD‐BirA*‐Myc fusion in MDCK II cells where it was shown to colocalize with endogenous canine E‐cadherin. BioID identified ∼300 potential E‐cadherin proximal proteins, the five most abundant of which (four isoforms of catenin and plakoglobin) were previously known components of adherens junctions. Another abundant protein was LPP (lipoma‐preferred partner protein), a member of the zyxin protein family. LPP was also identified in the ZO‐1 screen described in this section, pointing to the possibility that it could play roles at both adherens and tight junctions and prompting a detailed analysis of the role of this protein in cell function 25.

For occludin (Ocln) and claudin‐4 (Cldn4), Anderson and colleagues 24 stably and inducibly expressed *N*‐terminal BirA* fusions of both proteins (Myc‐BirA*‐Ocln and Myc‐BirA*‐Cldn4) and a *C*‐terminal Ocln‐BirA*‐Myc fusion in MDCK II cells and confirmed their localization to tight junctions and lateral membranes. Consistent with this localization, BioID with Ocln and Cldn4 identified numerous tight junction and lateral membrane proteins, as well as many cell signaling and trafficking factors and less prominently, proteins with diverse other functions and those with no known function. Combined with the results obtained with ZO‐1 and E‐cadherin 25, 26, the Ocln and Cldn4 data 24 provides significant insights into the protein composition and organization of cell junctions and represents an unparalleled resource for future investigations.

Two further studies have appeared that apply the BioID concept to cell junctions, albeit with immunoblotting, rather than MS, being used to identify proteins labeled by BirA* 27, 28. Working with MDCK cells stably expressing a GFP‐BirA*‐α‐catenin fusion protein, Yamada and colleagues 27 took the imaginative approach of performing BioID in cells subjected to mechanical stretching. Increased biotinylation of myosin IIA is detected under stretched conditions compared to nonstretched controls, leading to the suggestion that myosin IIA is recruited to regions of cell–cell contact under stretched conditions only. Elsewhere, an interaction between MarvelD3, a transmembrane component of tight junctions, and the MEKK1 kinase 28 was detected by BioID. Both Myc‐BirA*‐MarvelD3 and MarvelD3‐BirA*‐HA fusions, stably expressed in MDCK cells, were used in these experiments, with the interaction being detected by immunoblotting for MEKK1 in biotinylated fractions.

### 2.5 Autophagy

The TBC1D14 protein is a negative regulator of autophagy in mammalian cells that associates with members of the TRAPP (trafficking protein particle) complex. Tooze and colleagues 29 adapted the standard BioID strategy to establish which of the TRAPP components is closest to TBC1D14. To achieve this, label‐free quantitative proteomics (specifically iBAQ, intensity‐based absolute quantification) 30 was used to quantify TRAPP subunits pulled down from Myc‐BirA*‐TBC1D14 cell extracts prepared under denaturing (1% SDS) and nondenaturing conditions. The results of these experiments established TRAPPC8 as the most proximal to TBC1D14 and led to the suggestion that TRAPPC8 mediates the TBC1D14–TRAPP complex interaction, a proposal that was confirmed by showing the interaction was disrupted in TRAPPC8 knockdown cells 29. To date, this study is alone in applying a quantitative proteomic approach to BioID data analysis (discussed further in Section 5).

### 2.6 Cell signaling pathways

The Hippo pathway plays an important role in controlling organ size in mammals through regulation of cell proliferation and apoptosis 31. The core of the pathway is a protein kinase cascade. Activation of the pathway causes the MST1 and MST2 kinases to phosphorylate and activate the LATS1 and LATS2 kinases, which then phosphorylate and effectively inactivate the transcriptional coactivators YAP1 and TAZ1 by ensuring their retention in the cytosol. When active in the nucleus, YAP1 and TAZ1 function to promote cell proliferation and inhibit apoptosis.

In order to define the mammalian Hippo interactome, Gingras and colleagues 32 performed BioID using 19 HeLa or HEK293 cell lines stably and inducibly expressing different Flag‐BirA* fusion proteins, including key pathway components such as the human MST1, MST2, and LATS1 and murine Lats2 kinases and the human YAP1 transcription factor. In total, BioID identified more than 300 proteins as potential components of the Hippo interactome, almost three times more than were identified in parallel experiments on a partially overlapping set of 21 Hippo pathway proteins performed by affinity purification 32, with the increased number of proteins identified by BioID appearing, at least in part, to reflect the ability of this method to overcome practical difficulties associated with low protein solubility and weak protein–protein interactions.

The cerebral cavernous malformation (CCM) complex has an essential function in cardiovascular development in endothelial cells but how this function is mediated is unclear. Using BioID Kahn and colleagues 33 established a direct link between CCM and the MEKK3 MAPK signaling pathway. To do this, MEKK3 was expressed from a lentiviral vector as an HA‐BirA*‐MEKK3 fusion in human hCMEC/D3 endothelial cells; biotinylated proteins identified by MS included the CCM complex members CCM2 and KRIT1.

In addition to being useful for studying protein kinases, BioID has also been used to study phosphoprotein phosphatase 2A (PP2A) function in liver metabolism with a view to identifying factors that interact with the PP2A regulatory subunit PPP2R5C 34. In this study, Myc‐BirA*‐PPP2R5C and PPP2R5C‐BirA*‐HA fusions were expressed in mouse Hepa1‐6 cells and biotinylated proteins identified by immunoblotting. This study also made use of a cotransfected catalytically inactive PP2A catalytic subunit in an effort to trap substrates that otherwise interact only fleetingly with PPP2R5C.

The calmodulin‐like CALML5 protein plays an important role as a regulator of epidermal differentiation. BioID analysis using functionally validated BirA*‐CALML5 fusion proteins in differentiated keratinocytes 35 identified a number of proteins including the 14‐3‐3 protein stratifin (SFN), mutations in which had previously been shown to impair epidermal differentiation in mice 36, 37. Subsequent BioID utilizing a BirA*‐CALML5 fusion in both normal and CALML5‐depleted differentiated keratinocytes identified a number of known SFN interactors, including at least one protein, EXPH5 (exophilin‐5), whose interaction with SFN is reduced in the absence of CALML5, thereby implying a role for CALML5 in modulating SFN–EXPH5 interactions.

### 2.7 Nonsense‐mediated mRNA decay

Nonsense‐mediated decay (NMD) is responsible for the turnover of mRNA transcripts that contain premature termination codons in eukaryotic cells 38. UPF1, UPF2, and UPF3 are interacting core NMD factors, with the RNA helicase UPF1 having a key role in recognition of premature termination codons. Additional proteins such as the SMG1 kinase, its regulators SMG8 and SMG9, the nuclease SMG6, and the SMG5‐SMG7 heterodimer are then involved in subsequent processing events.

In order to gain greater insight into the machinery of NMD, Mühlemann and colleagues 39 performed BioID with UPF1, UPF2, and SMG5 BirA* fusion proteins. Prior to undertaking this work, a comprehensive series of control experiments were carried out to confirm (i) that BirA* biotinylation occurred exclusively in vivo, prior to cell lysis, (ii) that each BirA* fusion protein (UPF1, UPF2, SMG5) was able to rescue the NMD defect of cells in which the corresponding endogenous protein had been depleted by RNA interference, and (iii) that the activity of the BirA* fusion proteins was comparable to that seen with equivalent nonpromiscuous BirA fusions, indicating that the extensive self‐biotinylation of the BirA* fusion proteins expected during the course of the experiment did not perturb protein function. BioID was then performed in 293T cells transiently expressing HA‐BirA* fusion proteins, with biotinylated proteins being identified by immunoblotting and MS. This approach led to the identification of a number of proteins previously known to interact closely with UPF1, UPF2, and SMG5, plus range of potential novel interactors such as the signaling adaptor molecule CRKL and the heat shock protein DNAJB1, both of which were biotinylated by all three BirA* fusions but not in the corresponding controls, as well as mRNA decapping factors and components of the translation machinery 39.

### 2.8 Ubiquitin‐mediated proteolysis: E3 ligases and deubiquitylating enzymes

SCF^β‐TrCP1^ and SCF^β‐TrCP2^ are related ubiquitin E3 ligases that play key roles in a number of important cell signaling pathways (including the Hippo pathway described in the previous section, but also the Wnt, Hedgehog, and NFκB pathways) by targeting proteins for proteasomal degradation 40. Identifying E3 ligase substrates by traditional biochemical means is particularly challenging as E3 ligase–substrate interactions are often low affinity and/or transient and of course, result in degradation of the substrate. In their screen for SCF^β‐TrCP1/2^ substrates using BioID, Raught and colleagues 41 overcame the last of these three problems through the use of the proteasome inhibitor MG132 to protect ubiquitylated substrates from degradation. The F‐box proteins β‐TrCP1 and β‐TrCP2 were each stably and inducibly expressed in HEK293 cells as *N*‐terminal Flag‐BirA*‐β‐TrCP fusions and almost 300 proximal proteins identified with high confidence, including known components of the SCF^β‐TrCP1/2^ complexes such as SKP1, CUL1, and RBX1 (identified both in the presence and absence of MG132, as would be expected) as well as previously identified SCF^β‐TrCP1/2^ substrates such as β‐catenin, claspin, and the CDC25A and CDC25B phosphatases (identified only in the presence of MG132). Further analysis identified almost 80 additional proteins enriched in MG132‐treated fractions as potential SCF^β‐TrCP1/2^ substrates, including proteins representative of a broad range of functional categories. How many of these are authentic SCF^β‐TrCP1/2^ substrates remains to be seen, as Coyaud et al. 41 make the important point that stabilization of an individual substrate that is part of a larger protein complex (β‐catenin is one such example) will almost certainly lead to increased biotinylation of its interacting partners.

BioID has also been used as part of wider study by Sanyal and colleagues 42 to identify deubiquitylating enzymes involved in T‐cell receptor signaling in primary T lymphocytes and their substrates. BioID performed in activated and nonactivated human Jurkat cells using a Myc‐BirA*‐Usp12 fusion protein identified a number of potential Usp12 substrates, including several proteins previously reported to stabilize T‐cell receptors at the cell surface such as LAT and Trat1 42.

### 2.9 Mitochondrial proteasome

The protease ClpP and the AAA^+^ ATPase ClpX are components of the ClpXP proteasome complex 43 that is responsible for the degradation of misfolded proteins in the mammalian mitochondrial matrix. To identify potential substrates of ClpP, Schimmer and colleagues 44 performed BioID in mitochondria for the first time, stably expressing catalytically active or inactive BirA*‐ClpP fusion proteins in 293 T‐REx cells. Importantly, a BirA* fusion to the unrelated mitochondrial matrix protein ornithine transcarbamoylase was used as a control in these experiments, which ultimately led to the identification of ∼50 preferential ClpP interactors, including ClpX and a range of proteins implicated in mitochondrial metabolism. The demonstration that BioID can, like APEX 4, 5, be applied in the mitochondria opens up a range of possibilities for future studies of mitochondrial protein function.

### 2.10 Oncogenic transcription factors

Ewing's sarcoma is the second most common bone cancer in children and young people and is characterized by chromosomal translocation events that fuse the gene encoding the EWS protein with one encoding an Ets family transcription factor, most often Fli‐1 45. The resulting EWS‐Fli‐1 fusion is an oncoprotein that drives expression of a number of genes involved in cell proliferation and transformation and as such is an ideal target for therapeutic intervention 46. As part of a study aimed at gaining insights into the biochemical properties of the EWS‐Fli‐1 protein, Shiio and colleagues 47 performed BioID on HEK293T cells that had been transiently transfected with plasmid expressing a Myc‐BirA*‐EWS‐Fli1‐1 fusion protein. Over 350 potential proximal proteins were detected by this method. Further experiments confirmed the interaction of EWS‐Fli1‐1 with CIMPR, a protein involved in the endosome‐lysosome system, leading to the observation that EWS‐Fli1‐1 is turned over by a lysosome‐dependent mechanism and the suggestion that targeting EWS‐Fli1‐1 for degradation might be an effective therapy for Ewing's sarcoma 47.

Like EWS‐Fli‐1, c‐MYC is an oncogenic transcription factor. c‐MYC regulates the expression of a number of genes, including genes involved in cell growth, cell cycle progression, differentiation, and apoptosis, and deregulated c‐MYC expression drives tumor formation 48. To identify c‐MYC proximal interactors in vivo, Raught and colleagues 49 introduced a plasmid capable of expressing a Flag‐BirA*‐c‐MYC fusion under the control of a tetracycline‐inducible promoter into HEK293 cells. The functionality of the Flag‐BirA*‐c‐MYC protein was assessed in these cells by inducing protein expression and confirming the ability of the fusion protein to interact with the c‐MYC binding partner Max and its ability to activate transcription of the target gene *NCL* and repress transcription of *CDKN1A*. Cells expressing the Flag‐BirA*‐c‐MYC fusion (or Flag‐BirA* alone as a control) were then injected subcutaneously into immunodeficient NOD/SCID mice. Expression of the Flag‐BirA*‐c‐MYC fusion was induced by feeding the animals with tetracycline, resulting in tumor formation. The mice were then injected with biotin over 2 days, before tumor samples were processed for BioID. For comparison, BioID was also performed on cultured cells expressing the Flag‐BirA*‐c‐MYC fusion (or Flag‐BirA* alone). These approaches led to the identification of >100 high‐confidence c‐MYC proximal interactors present in both cultured cells and xenografts, including over 30 previously known interactors such as Max 49. Novel interactors included proteins involved in, for example, transcription, RNA processing, and chromatin remodeling.

### 2.11 Histones H2B and H3

In addition to the chromatin‐associated proteins described in the previous section, BioID has also been applied the core histones H2B and H3 50. To achieve this, the H2B and H3 proteins were stably expressed in HEK‐293 cells as H2B‐ and H3‐BirA*‐Flag fusions. Parental HEK‐293 and cell lines expressing GFP‐BirA*‐Flag and nuclear localization signal (NLS)‐BirA*‐Flag were used as negative controls. Using the most stringent filtering parameters, 210 interaction partners were identified for H2B and 90 for H3B, including various histone chaperones, chromatin remodeling complexes, mitotic proteins, etc., with considerable overlap between the two datasets. These numbers for BioID represent a two to three times increase in the number of significant interactors for each protein compared to earlier affinity purification studies from the same lab 51. Strikingly, few interactors were identified by both BioID and affinity purification approaches (21 out of 210 BioID hits for H2B, ten out of 90 for H3) and for those proteins identified by both approaches, the evidence from spectral counts suggests that the different purification methods favor certain proteins over others. Taking histone chaperones as an example, CHAF1A/CHAF1B is found with H3 in greater amounts with BioID than affinity purification, NAP1L1/NAP1L4 is found with H2B in greater amounts with affinity purification than BioID, and ASF1A/ASF1B is found with H3 in similar amounts with BioID and affinity purification 50. The reason for this behavior is unclear. However, it is clear that there is much to be gained from using the two approaches in parallel, rather than relying on a single method.

### 2.12 Mediator

Mediator is a large (∼30 subunits, >1 MDa) multiprotein complex that plays an essential role in both initiation and elongation stages of eukaryotic transcription by bridging RNA polymerase II, DNA‐binding transcriptional regulators, the general transcription factors and transcription elongation factors 52. Structurally, the Mediator complex comprises four distinct modules (head, middle, tail, and kinase) whose subunit composition and arrangement with respect to one another has been characterized by a combination of techniques including cryo‐EM and partial crystallography. In addition to histones H2B and H3 described in Section 2.11, Lambert et al. 50 performed BioID on three mediator subunits—Med20, Med4 and Med23, components of the head, middle and tail modules, respectively—and generated individual HEK‐293 cell lines stably expressing *C*‐terminal BirA*‐Flag fusions with these proteins. Parental HEK‐293 and GFP‐BirA*‐Flag and NLS‐BirA*‐Flag expressing cell lines were used as negative controls. BioID of the Med23 and Med4 proteins identified 25 and 20 Mediator subunits (from all four constituent structural modules), respectively, as well as a range of other potential interactors. BioID of Med20 identified six Mediator subunits only, from the middle and tail modules, but it should be noted that the level of Med20‐BirA*‐Flag expression was low, which may explain the low rate of return from this experiment. Interestingly, BioID with Med4 (but not Med20 nor Med23) identified a number of centrosome components and Lambert et al. 50 were subsequently able to demonstrate by immunofluorescence microscopy that the Med4 protein colocalizes with the centrosomal markers centrin and pericentrin throughout the cell cycle. The biological significance of this novel observation remains to be elucidated but it does underline the power of the BioID approach in uncovering hitherto unexpected connections between proteins. As with histones H2B and H3, Lambert et al. 50 also performed AP‐MS (affinity purification followed by MS) of Med20, Med4, and Med23, finding only limited overlap between AP‐MS and BioID datasets and emphasizing once again the importance of taking multiple experimental approaches to local interactome mapping.

### 2.13 HMG CoA reductase and Schnyder corneal dystrophy

Schnyder corneal dystrophy is a human genetic disease that results in blindness due to a buildup of cholesterol in the corneas. Schnyder corneal dystrophy is caused by dominant mutant forms of UBIAD1 (UbiA prenyltransferase domain‐containing protein‐1) but how perturbation of UBIAD1 function leads to cholesterol buildup is unclear. Using BioID, DeBose‐Boyd and colleagues 53 recently demonstrated an interaction between UBIAD1 and HMG CoA reductase, an enzyme that catalyzes a rate‐limiting step in cholesterol production in the ER. To do this, the membrane domain (residues 1–346) of the hamster HMG CoA reductase was expressed in human HEK293 cells as an HSV‐reductase‐BirA* fusion under the control of the cytomegalovirus promoter. Further analysis revealed that UBIAD1 binding acts to protect the reductase from degradation, thus perturbing the normal control of reductase activity by negative feedback, resulting in cholesterol accumulation.

## 3 BioID in unicellular organisms

### 3.1 Trypanosoma brucei

The unicellular eukaryotic parasite *T. brucei* is the causative agent of human African trypanosomiasis (sleeping sickness), a neglected tropical disease that constitutes a serious health risk to some 60 million people in sub‐Saharan Africa. Three BioID studies using *T. brucei* have been reported, two of which 54, 55 focused on the biology of the kinetoplastid flagellum, which plays a vital role in parasite motility.

The first of these studies aimed to characterize the bilobe, a discrete cytoskeletal structure that partially overlaps with the flagellum attachment zone (FAZ) at the posterior end of the cell and in which the TbMORN1 protein is localized. To perform BioID on the bilobe, TbMORN1 was stably expressed as a Myc‐BirA*‐TbMORN1 fusion protein in procyclic form *T. brucei* parasites 55. The level of Myc‐BirA*‐TbMORN1 expression was broadly similar to endogenous TbMORN1 and the fusion protein localizes to the bilobe, suggesting that a degree of functionality is retained despite the addition of the Myc‐BirA* sequences. Incubation of Myc‐BirA*‐TbMORN1 expressing cells with biotin also results in specific labeling of the bilobe. Twelve biotinylated proteins present in the detergent‐insoluble cytoskeletal fraction were identified by MS, including TbMORN1 itself, TbLRRP1, known to colocalize with TbMORN1, and ten previously uncharacterized proteins of wholly unknown function. Of these ten, nine were subsequently shown to colocalize with or be adjacent to TbMORN1, either as part of the bilobe or FAZ 55.

The second *T. brucei* BioID study 54 utilized an SAS‐4‐BirA*‐HA fusion protein, SAS‐4 being a component of the basal body, the structure that nucleates kinetoplastid flagellar assembly. This study identified 20 putative SAS‐4 interactors, including four known FAZ components, three novel FAZ components, and five proteins that had previously been identified as novel components of the bilobe by TbMORN1 BioID 55.

The third BioID study in *T. brucei* 56 focused on the polo‐like kinase homologue TbPLK. This protein localizes to the basal body, bilobe, FAZ and flagellar connector, and BioID using Myc‐BirA*‐TbPLK identified <50 candidate proximal proteins, many of which associated with these structures, including five proteins that has previously been identified in the TbMORN1 screen 55. The effectiveness of BioID in *T. brucei* has the potential to revolutionize the study of this and related kinetoplastid organisms by offering a simple and reliable method for forward screens for novel interactors.

### 3.2 Toxoplasma gondii

*Toxoplasma gondii* is an obligate intracellular parasite found in 30–50% of the global population 57. In healthy adults, infection with *T. gondii* is generally asymptomatic, although mild flu‐like symptoms are possible in the first few weeks after exposure. In immunocompromised individuals, such as acquired immunodeficiency syndrome (AIDS) patients, or those undergoing treatment with immunosuppressant drugs, infection can result in a more serious disease, even death. There is an urgent need to understand the biology of the parasite better with a view to developing effective treatments.

#### 3.2.1 Inner membrane complex

The inner membrane complex (IMC) is a Golgi‐derived double‐membrane structure that underlies the plasma membrane, and which is unique to apicomplexan organisms such as *T. gondii* and the malaria parasite *Plasmodium falciparum* 58. The IMC plays important roles in motility, host cell invasion, and replication in *T. gondii*, making it an attractive target for therapeutic intervention. Some progress has been made toward defining the protein composition of IMC but the nature of the organelle presents challenges to conventional interactome mapping approaches.

To circumvent these problems, Chen et al. 59 performed BioID in *T. gondii* using an ISP3‐BirA*‐HA fusion protein. ISP3 is a key component of the IMC membrane sacs. The ISP3‐BirA*‐HA fusion was stably expressed from the ISP3 promoter and localized within the IMC in a manner indistinguishable from the endogenous ISP3 protein. BioID performed on parental and ISP3‐BirA*‐HA expressing cells in parallel led to the identification of a number of putative ISP3 interactors, including a number of proteins that had been previously shown to associate either with IMC membranes or with the cytoskeletal network underlying the IMC, as well as uncharacterized proteins. Several of the uncharacterized proteins were shown by immunofluorescence to localize to the central and basal subcompartments of the IMC and others to localize to the apical cap (AC) structure. The AC is involved in the entry of the parasite into the host cell. To identify additional AC components, one of these novel proteins (AC2) was expressed as an AC2‐BirA*‐HA fusion for BioID, leading to the identification of several new AC components 59.

#### 3.2.2 TgCDPK3 kinase substrates

TgCDPK3 is a calcium‐dependent protein kinase (CDPK) that is critical for egress of *T. gondii* parasites from infected cells and subsequent spread through the infected individual. To identify potential TgCDPK3 substrates by BioID, Gaji et al. 60 stably expressed a TgCDPK3‐BirA*‐HA fusion under the control of the *Tgcdpk3* promoter in a *Tgcdpk3* mutant strain, allowing them to unambiguously demonstrate the functionality of the fusion protein in an egress assay prior to performing biotin labeling. BioID was performed in parallel with a *Tgcdpk3* mutant strain expressing a TgCDPK3‐HA fusion protein as control. Fourteen proteins were detected in the TgCDPK3‐BirA*‐HA strain, seven of which, including the myosin A TgMyoA, had previously been identified as being differentially phosphorylated in wild‐type and *Tgcdpk3* mutant cells, underlining their credentials as TgCDPK3 substrates. Further analysis of TgMyoA phosphorylation by TgCDPK3 demonstrated the importance of this protein for parasite egress. The success of these studies in *T. gondii* highlights the applicability of BioID for the exploration of diverse cellular processes in unicellular organisms, possibly leading to the identification of new therapeutic targets.

### 3.3 Dictyostelium discoideum

The amoeba *D. discoideum* is a widely used model for a variety of cell biological processes, including cellular differentiation, signal transduction, and chemotaxis 61. As part of their analysis of the nuclear lamina in *D. discoideum*, Gräf and colleagues 62, 63 have performed BirA* labeling studies with the NE81 lamin protein. As discussed previously (Section 2.1), lamin proteins form insoluble networks under physiological conditions and so present a significant challenge to established methods of protein–protein interaction screening.

To apply the method to *D. discoideum*, a BirA*‐NE81 fusion was expressed under the control of the regulatable *actin6* promoter, the BirA* ORF having first been codon‐optimized to match the AT‐rich nature of *D. discoideum* coding sequences. Plasmids expressing the BirA*‐NE81 fusion were integrated in single or multicopy into the *D. discoideum* genome and high‐level fusion protein expression was induced by growth in axenic medium. Following purification of biotinylated proteins, immunoblotting revealed proximity labeling by BirA*‐NE81 of endogenous NE81, the lamin‐binding protein SUN1 63, and the MAN1‐like protein Src1 62. Additional proteins specifically biotinylated in the presence of BirA*‐NE81, but not in the control cells expressing BirA* alone, were also visualized in these experiments but as yet no analysis of these potentially novel interactors has been published.

## 4 Wider applications of BioID

### 4.1 Viral infection

Human immunodeficiency virus (HIV) is the causative agent of AIDS. HIV infects helper T cells (specifically CD4^+^ T cells), macrophages, and dendritic cells and brings about a progressive failure of the immune system that results in the infected individual becoming increasingly susceptible to opportunistic infections and cancer. Approximately 40 million people worldwide are currently living with HIV, mostly in sub‐Saharan Africa, with over 1 million dying per year. The Gag precursor polyprotein and the mature proteins produced when this is proteolytically processed play important roles at various points in the HIV‐1 replication cycle 64. Processing of the Gag precursor yields the matrix (MA), capsid, nucleocapsid, and p6 proteins.

In order to better understand the roles of these proteins in the infection process, two groups have performed independent BioID studies on Gag using very different experimental strategies 65, 66. Mouland and colleagues 66 took the relatively straightforward route of transiently expressing a Myc‐BirA*‐Gag fusion protein in both Jurkat (T lymphocytes) and HeLa cells. After confirming by immunofluorescence that the protein was correctly localized, BioID was performed on Myc‐BirA*‐Gag expressing Jurkat cells, with cells expressing Myc‐BirA* alone used as a control. This led to the identification of ∼50 proteins proximal to Myc‐BirA*‐Gag, including proteins involved in translation and innate immunity. For two of these proteins (DEAD‐box RNA helicase DDX17 and ribosomal protein S6), interactions were confirmed by coimmunoprecipitation 66.

In contrast, Barklis and colleagues 65 performed a more elaborate study that compared data obtained using wild‐type Gag precursor with that from an internally deleted Gag lacking most of the MA domain. For these experiments, Myc‐BirA* was placed between the MA and capsid domains of the wild‐type precursor Gag protein, rather than at the *N*‐terminal end, and expressed from proviral expression constructs. Around 50 proteins were found to be biotinylated in cells expressing the wild‐type BirA*‐Gag protein and/or the ∆MA‐BirA*‐Gag version, including translation factors, cytoskeletal proteins, membrane proteins, and RNA‐processing factors, over half of which has previously been demonstrated or implied to interact with HIV‐1. Comparison with the data of Le Sage et al. 66 reveals that only one protein (Lyric/AEG‐1) was found in both screens, presumably reflecting the different experimental setups.

In addition to analysis of Gag function, BirA* has also been used as a minor part of an extensive study aimed at dissecting the role of the Vpu protein in HIV‐1 biogenesis 67. In this study, Vpu‐myc‐BirA* was shown to biotinylate the clathrin adaptor protein AP‐1 in 293T cells expressing the host cell antiviral protein tetherin, which is antagonized by Vpu.

Beyond HIV‐1, BioID has also been used to study interactions between the Epstein–Barr virus latent membrane protein 1 (LMP1) protein and actinin 1 (Actn1) 68. LMP1 is a multifunctional membrane protein that is required for establishment of latent Epstein–Barr virus infection through the activation of diverse signaling pathways. For BioID experiments, wild‐type and mutant forms of LMP1 were expressed from retroviral vectors in C33A cervical carcinoma cells as LMP1‐BirA* fusions and biotinylated fractions immunoblotted with anti‐Actn1 antibodies, leading to the conclusion that LMP1 interacts with a high molecular weight (potentially ubiquitylated) isoform of Actn1 68.

### 4.2 Bacterial infection

The Gram‐negative bacterium *Chlamydia psittaci* (also known as *Chlamydophila psittaci*) is a widely distributed avian pathogen that can be transmitted to humans to cause a variety of flu‐like symptoms leading to potentially life‐threatening pneumonia 69. *Chlamydia psittaci* is an obligate intracellular parasite. Outside the host cell, *C. psittaci* cells are found as metabolically dormant but highly infective particles called elementary bodies (EBs) that can be taken up into the host cell by phagocytosis, resulting in the formation of pathogen‐specific organelles called inclusions. Within inclusions, EBs differentiate to actively replicating but noninfectious particles known as reticulate bodies (RBs). Mature RBs then differentiate back to EBs which, following release from the host (often through host cell lysis), go on to infect other cells within the original host or to be transmitted to other individuals.

The *C. psittaci* SINC protein is one of a number of effector proteins that are expressed in the RB and secreted from there into the host cell cytoplasm, a process that involves translocation across successive bacterial and endosomal membranes. Ultimately, SINC enters the nucleus of infected cells where it localizes to the NE and the INM and nuclear lamina in particular 70. Remarkably, SINC localizes to the same sites in adjacent noninfected cells also, although how this occurs is unclear as yet.

To better understand the subcellular localization and role of SINC, Bavoil and colleagues 70 stably expressed a Myc‐BirA*‐SINC fusion protein in HEK293 cells for BioID, after first confirming that the protein localized correctly to the NE when transiently expressed in HeLa cells. As expected, BioID identified several INM and nuclear pore proteins, including lamin B1, lamina‐associated protein 1 (LAP1), emerin, and MAN1, all four of which had previously been identified 6 by BioID of LaA (discussed in Section 2.1). In total, 50 proteins proximal to the Myc‐BirA*‐SINC fusion were identified in two independent but identical BioID experiments, although it should be noted that only around half of these were found in both, including the four mentioned above 70. The remaining high‐confidence proximal proteins include factors involved in nuclear import, ER structure and function, vesicle trafficking, innate immunity, and signaling, opening up new avenues of investigation of the role of SINC during *C. psittaci* infection.

Biotin labeling by BirA* has also been used to study the mechanism of host cell protein localization to the inclusion of a second chlamydial species, *Chlamydia trachomatis*, the causative agent of trachoma and the most common bacterial sexually transmitted infection. Moore and colleagues 71 used BioID in *C. trachomatis*‐infected HeLa cells to demonstrate that targeting of the host cell SNARE protein syntaxin 6 to the inclusion was dependent on the presence of the signal sequence YGRL, visualizing full‐length and truncated syntaxin 6 localization using a fluorescent streptavidin conjugate. Biotinylated protein pull‐down and MS was not performed in this study 71.

## 5 Technical considerations

### 5.1 Design and expression of the BirA* fusion protein

The starting point for BioID is the expression of a fusion protein between the protein of interest and the BirA* enzyme. BirA* has a molecular mass of 35.4 kDa (∼10 kDa larger than GFP for example) and is comprised of three distinct domains, with the active site of the enzyme being located in the central domain 72, 73. As described in the previous sections, both *N*‐ and *C*‐terminal BirA* fusions have been used for BioID studies, with constructs also typically incorporating an antibody epitope tag (HA, Myc, or Flag) onto the distal end of the BirA* protein for convenient immunochemical analysis of fusion protein abundance and localization. The optimal location (*N*‐ or *C*‐terminal) for the attachment of BirA* will depend on the target protein and for elongated proteins such as CEP152 or ZO‐1 discussed above 21, 26, the spatial specificity of biotin labeling may mean that valuable additional information can be gained by performing independent BioID experiments with *N*‐ and *C*‐terminal fusions in parallel. Under certain circumstances, such as in the analysis of the HIV‐1 Gag precursor protein interactome 65, there may also be advantages to placing BirA* internally in the protein of interest, although this clearly requires prior knowledge of the domain structure of target protein to ensure that BirA* insertion does not disrupt protein structure and function and potentially invalid the results obtained from BioID.

Ideally, the BirA* fusion should be expressed in cells at a similar level to the endogenous target protein, but testing expression levels normally requires specific antibodies directed against the target protein, which are not always available. Even if high levels have no detrimental effects on cell behavior, overproduction of the BirA* fusion protein brings with it the increased possibility of mislocalization, nonspecific biotin labeling, and false positive protein identifications. In most of the BioID studies on mammalian proteins described in the previous sections, sequences encoding the BirA* fusion are stably integrated into the genome of cultured cells (typically HEK293 or its derivatives) with expression under the control of a heterologous promoter. Stable integration of the expression plasmid was also the method of choice for BioID in *T. brucei* 55, *T. gondii* 59, 60, and *D. discoideum* 63, with expression of the *T. gondii* BirA* fusions being driven in both cases by endogenous promoters. Only in a minority of cases has BioID been performed following transient cell transfection (see for example 21, 47).

For future BioID experiments, a novel endogenous gene tagging technique for mammalian cells could be utilized where a multifunctional integrase tag is inserted in‐frame of a gene locus of interest 74. The tag allows an efficient recombination‐based integration of any functional cassette by utilizing the Bxb1 integrase enzyme. The developers of this method used it to perform a BioID study of the proximal proteome of the epigenetic factor TET1 by inserting the BirA* cassette into *Tet1* locus, such that the BirA*‐Tet1 fusion protein was expressed at normal endogenous levels. In the future, this method would allow physiological conditions to be maintained during BioID experiments and potentially detrimental effects due to overexpression of the fusion protein would be avoided 74.

### 5.2 Localization and function of the BirA* fusion

Once the appropriate cell line is constructed, it is important to confirm the subcellular localization of the BirA* fusion protein by immunofluorescence microscopy with antibodies raised against either the epitope tag, BirA (anti‐BirA antibodies are commercially available from several suppliers) or the target protein. Correct localization does not guarantee that the protein of interest will have retained full function, of course, but whether this is important will depend on the research question being addressed. If the main aim is to explore the subcellular environment of the protein of interest, then correct localization of the fusion protein should be sufficient. However, if the protein of interest possesses an enzymatic function that is required for the interactions with the other proteins, then these interactions will not be detected by BioID if the protein is inactive. Accordingly, it seems wise to validate the function of the protein wherever possible. The methods available to test functionality depend on the protein under study. In *T. gondii* for example, BioID with the TgCDPK3‐BirA*‐HA fusion was performed in *Tgcdpk3* mutant cells only after the function of the fusion protein was tested in a parasite egress assay 60, whereas in mammalian cells, BioID with the Flag‐BirA*‐CEP120 fusion was performed only after it was shown that the fusion could rescue the phenotype of cells treated with a CEP120 siRNA 22. The latter approach will be applicable to many potential BioID targets but whether it finds wide acceptance in the community remains to be seen.

### 5.3 Data analysis

MS data from BioID experiments comprise a large list of potential interactome components. Validating the entire list of potential interactors may not be feasible, yet certain proteins have to be selected for further analysis. The studies discussed in this review have often chosen proteins that were isolated at highest abundance from MS data. The highest abundance is a semiquantitative approach and may not reflect the true strength of association between two proteins 11, but it is an appropriate starting point.

Perhaps the ideal way to select for high‐confidence interactors would be by combining BioID with quantitative proteomics techniques such as SILAC 75, iTRAQ 76, or SWATH 77. The power of quantitative proteomics combined with proximity‐dependent labeling is well illustrated by the APEX studies conducted by Ting and colleagues 4, 5. These researchers found that performing SILAC‐based MS in APEX experiments allowed elimination of background proteins and revealed the list of neighboring proteins with higher confidence. Therefore, quantitative proteomics approach is likely to improve BioID experiments also.

An important step at eliminating nonspecifically biotinylated proteins from BioID experimental results is performing appropriate controls. The issue of control design has been extensively discussed by Gingras and colleagues 50 and will be only briefly mentioned here. Generally, in the BioID studies performed to date, one of two types of negative control has been performed, using either the parental cell line or using cells expressing an epitope‐tagged BirA* protein at the same level as the target protein BirA* fusion. Lambert et al. 50 themselves trialed a GFP‐BirA*‐Flag fusion in their study of the histone H2B and H3 and Mediator subunit interactomes, as well as NLS‐BirA*‐Flag fusion, and reached the conclusion that having multiple types of control was more useful than having multiple replicates of a single type. The proteins identified from controls could be subtracted from the experimental results to narrow down to a list of potential interactors with higher confidence.

## 6 Future prospects

One potential obstacle to the application of BioID is the relatively large size (35.4 kDa) of the *E. coli* BirA* protein. As noted previously, fusion of BirA* with the target protein can result in impaired protein localization and function 78. In an effort to mitigate this problem, Roux and colleagues 78 investigated the potential of BirA protein from the thermophilic bacterium *Aquifex aeolicus* for BioID studies 78. The *A. aeolicus* protein is the smallest known BirA protein, significantly shorter than *E. coli* BirA (223 versus 321 amino acids) and lacking the latter's *N*‐terminal DNA binding domain. When tested in comparison with the standard *E. coli* BirA* enzyme, *A. aeolicus* BirA* (BirA‐R40G, where R40G is equivalent to the promiscuity‐conferring R118G mutation in the *E. coli* BirA sequence) required significantly less biotin for maximal activity and allowed better localization of at least one fusion protein. When fused to the NPC protein Nup43 (see Section 2.2) in HEK293 cells, the *A. aeolicus* enzyme appeared to display enhanced biotinylation activity and a potentially larger labeling radius. Increasing the distance between Nup43 and the *A. aeolicus* BirA* by linker insertion led to a further increase in labeling radius without loss of labeling specificity. It seems highly likely that the *A. aeolicus* BirA* enzyme (designed BioID2) will replace the *E. coli* prototype in future BioID studies 78.

## 7 Concluding remarks

In the 4 years from the introduction of BioID 6, the method has already made significant contributions to mapping local interactomes relevant to a wide range of biological processes, both in mammalian cells and in single‐celled eukaryotes (Table 1). The strength of BioID lies in its ability to identify proteins that interact indirectly or weakly with the protein of interest or which are located in the vicinity of the protein of interest without making either a direct or indirect interaction. BioID has also proved especially relevant to the study of insoluble, inaccessible, or low‐abundance structures such as the nuclear lamina 6, NPC 12, or centrosome 20, 21, 22 and can provide important information on subunit arrangement within large protein assemblies 12. BioID can also be used to identify transient protein–protein interactions, such as those between enzymes and their substrates 32, 41. Taken together, these attributes guarantee BioID methodology a vital place in the proteomics toolbox and highlight its potential to make continued major contributions to local interactome mapping in diverse organisms.

**Table 1:** Applications of BioID

	Section		BirA*‐tagged proteins	Reference
*Mammalian cells*				
	2.1	Nuclear lamina	LaA	6
			Progerin	16
			Lamin B1 (LMNB1), macroH2A1	17
	2.2	NPC	Nup53 (Nup93 complex component), Nup43, Nup85, Nup107, Nup133, and Nup160 (all Y‐complex components)	12
	2.3	Centrosome and centrosome‐cilium interface	CEP120	22
			PLK4, CEP192, CEP63, CEP152, CPAP, CCDC67, CCDC14, and KIAA0753.	21
			58 bait proteins	20
	2.4	Cell junctions	ZO‐1	26
			E‐cadherin	25
			Occludin, Claudin‐4	24
			MarvelD3	28
			α‐catenin	27
	2.5	Autophagy	TBC1D14	29
	2.6	Signaling pathways	Hippo pathway: 19 bait proteins	32
			MEKK3	33
			PPP2R5C	34
			CALML5, SFN	35
	2.7	Nonsense‐mediated mRNA decay	UPF1, UPF2, SMG5	39
	2.8	Ubiquitin‐mediated proteolysis	β‐TrCP1, β‐TrCP2	41
			Usp12	42
	2.9	Mitochondrial proteolysis	ClpP	44
	2.10	Oncogenic transcription factors	EWS‐Fli‐1 fusion protein, c‐MYC	47, 49
	2.11	Nucleosome	Histones H2B and H3	50
	2.12	Mediator complex	Mediator subunits Med4, Med20, Med23	50
	2.13	Schnyder corneal dystrophy	HMG CoA reductase, membrane domain	53
	‐	Cytokinesis	ULK3 (Unc‐51‐like kinase)	79
*Unicellular organisms*				
*Trypanosoma brucei*	3.1	Bilobe/hook complex	TbMORN1	55
			TbSAS‐4	54
			TbPLK	56
*Toxoplasma gondii*	3.2	IMC	ISP3, AC2	59
			TgCDPK3	60
*Dictyostelium discoideum*	3.3	Nuclear lamina	Lamin NE81	62, 63
*Host‐pathogen systems*				
Viral infection	4.1	HIV‐1	Gag polyprotein	65, 66
		HIV‐1	Vpu	67
		EBV	LMP1	68
Bacterial infection	4.2	*Chlamydia psittaci*	SINC	70
		*Chlamydia trachomatis*	Syntaxin 6	71

EBV: Epstein–Barr virus.
